# Older people’s experiences of oral health and assisted daily oral care in short-term facilities

**DOI:** 10.1186/s12877-021-02281-z

**Published:** 2021-06-27

**Authors:** Susanne Koistinen, Katri Ståhlnacke, Lena Olai, Anna Ehrenberg, Eva Carlsson

**Affiliations:** 1 School of Health and Welfare, Dalarna University, Falun, Sweden; 2 Department of Odontology, Dental Hygienist Education, Umeå University, Umeå, Sweden; 3 Dental Research Department, Postgraduate Dental Education Center, Örebro, Sweden; 4 Department of Public Health and Caring Sciences, Family Medicine and Preventive Medicine, Uppsala University, Uppsala, Sweden; 5 Faculty of Medicine and Health, University Health Care Research Center, Örebro University, Örebro, Sweden

**Keywords:** Daily oral care, Inductive content analysis, Older people, Oral health, Qualitative interviews, Short-term care

## Abstract

**Background:**

Older people’s oral health has improved, and many retain their natural teeth throughout their life. However, their daily oral care can be more difficult because of compromised general health and the reduced capacity for self-care that often comes with old age. More knowledge is needed about how older people view their oral health and oral care. The aim of this study was to describe how older people in short-term care experience their oral health and daily oral care.

**Method:**

A descriptive, qualitative study was performed through interviews with 14 older people (74–95 years) recruited from short-term care units in two Swedish regions. Data were analysed using inductive content analysis.

**Results:**

The findings are described in one main category, three categories and nine sub-categories. The main category was *Adapting to a changed oral condition while striving to retain independence*. The first category, *Wanting to manage daily oral care independently*, contained three subcategories: Having always brushed my teeth without help, Being satisfied with my mouth and teeth, and Having to accept help if necessary. The second category, *Acceptance of changes in oral condition*, had three subcategories: Difficulty in chewing and swallowing, Difficulty with tooth brushing, and Not considering a dentist visit to be worth the cost. The third category, *Barriers to receiving assistance from staff*, had three subcategories: Staff lacking the time to help, Not wanting to be a burden, and Lack of confidence in staff’s knowledge.

**Conclusions:**

The participants were generally satisfied with their oral health despite an expressed need for dental treatment. Daily oral care was something they wanted to manage themselves, and they had a strong desire to stay independent for as long as possible. Closer collaboration between dental and health care staff is necessary in order to implement clinical practice guidelines for oral health care and increase nursing staff’s attention towards older peoples’ oral health.

**Supplementary Information:**

The online version contains supplementary material available at 10.1186/s12877-021-02281-z.

## Background

Good oral health is important for older people’s well-being and quality of life, and is essential for healthy ageing [1]. However, with increasing age, declining health, and dependence on care, older people are more likely to develop poor oral health [2].

The number and proportion of older people is steadily increasing in most populations worldwide [3]. For example, the proportion of people 80 years and older, often referred to as the oldest old, is expected to increase by 50% in Sweden over the coming years [4]. Also, a growing number of older people in need of care are expected to increase as the population continues to age [5, 6]. The oldest old are often described as a frail and multi-morbid group [7]. For the individual, frailty increases vulnerability due to diminished strength, endurance, and physiological function [8].

Various forms of transitional care have been developed internationally, including intermediate care, geriatric rehabilitation units, home rehabilitation, and care planning units [9–11]. In the Swedish context, one such support organization for older people are named short-term care (STC). It is optional for the municipalities to provide such care, and there are no national guidelines for STC for older people [12]. STC staff include nurse aides, licensed practical nurses, registered nurses, occupational therapists, physiotherapists, and managers [13]. Some units have a rehabilitation profile, while others provide care for a mix of purposes such as rehabilitation, waiting for special accommodation, respite care, and even end-of-life care. The term STC can be misleading, as length of stay might exceed several months [12]. Approximately 9000 older people per year are admitted to Swedish STC [14].

The STC milieu is characterized by transition. From a physical perspective, the STC might be one station on the person’s journey from hospital or from home to special accommodation for older people [9]. From an existential perspective, this might be experienced as a crisis caused by acute illness that impacts further life, as a result of deteriorating chronic illness, or as a step closer to the end of life [10, 11, 15]. Therefore, for many it is an uncertain and worrying period in their life, not knowing if they will be able to continue living at home or will have to move to special accommodation [12].

In Sweden, older people in need of extensive care from the municipality have the right to a free-of-charge oral health assessment on an annual basis, in their home or special accommodation. Oral health care education is also offered to nursing staff to support them in assisting older people with daily oral care. Those who are entitled to this assessment also receive necessary dental treatment on the same economic basis as medical treatment [16].

Oral health is an important aspect of older people’s health and well-being. In general, oral health in Europe has improved, which has led to an increased number of older people retaining their natural teeth throughout their life [17]. In Sweden, approximately 60% of people aged 80–89 years have 20 or more teeth [18]. However, good oral health is more common among healthy older people than among frail persons in need of care in special accommodation [19, 20]. Further, poor oral health may have a negative impact on quality of life, nutritional status, and general health status [21, 22]. Frail individuals have a higher risk of developing poor oral health due to limitations in the ability to perform self-care and difficulties in visiting dental health care [2]. The reduced capacity for self-care may be due to mental and physical disability and various chronic diseases [23]. Thus, oral hygiene with daily removal of dental plaque by tooth brushing is essential to reduce the risk of dental diseases [24, 25]. Assisted daily oral care, including brushing of teeth or cleaning of dentures are often inadequate in nursing homes [26–28], although residents who are unable to perform activities of daily living should be given necessary assistance with daily oral care [29].

Most published studies about older people’s oral health have had a quantitative approach. For example, a Swedish study showed that 50% of people in STC were dependent on help with activities of daily living. However, only about 20% of them received assistance with oral care, even though clinical assessments made by registered dental hygienists showed that many more of them were in need of this [30]. An earlier Swedish study found impaired oral health status and a need for help with oral care among people who were dependent on help from others with activities such as bathing and dressing [31]. As the oldest old often suffer from multi-morbidity, frailty, and other health problems, the probability of needing assistance or care increases [32]. Therefore, older people who need support in their daily activities are also likely to need assistance with daily oral care [33].

There is insufficient knowledge about how older people view their oral health and oral care [34]. Qualitative studies exploring such experiences are scarce, and to our knowledge all such studies to date have involved participants living in ordinary homes or in special accommodation [35–37]. One such study, which reported on the perspectives of residents in a Canadian nursing home, found that having clean teeth was important to the participants, but as frailty increased, the importance of the mouth and the teeth decreased [38]. A recent Swedish study exploring older nursing home residents’ experiences of receiving assistance with bodily care found that these residents wished to be independent and care for their own bodies for as long as possible, and that they did not want to be a burden to the nursing staff [39].

Since providing bodily care involves handling of body fluids such as urine, sweat and blood it might be perceived as distressing and breaching personal integrity. Bodily care inevitably involves the nursing staff touching the bodies of older people and often performing a task that the older person would prefer to perform on their own and in private, such as brushing teeth and washing the genital [40]. There are several common barriers which hinder nursing staff in assisting older people with oral care. The older person may not want to accept the assistance that is offered, and nursing staff may lack knowledge, education, or training in providing oral care [41]. In addition, nursing staff have expressed that being able to take care of one’s own oral care is strongly associated with personal integrity and self-esteem [42]. Nursing staff have also reported not wanting to nag residents who decline assistance with oral care [43]. Older people’s motivation and capacity to prevent oral disease may be lost as frailty increases [37], for example because they want to use their energy for things other than maintaining their oral health [44]. Another reason to decline assistance with daily oral care might be the perception that one’s oral health is satisfactory. Koistinen and colleagues (2019) found that 85% of the older people from 36 Swedish STC units were satisfied with their oral health, even though clinical assessment by registered dental hygienists showed that 77% of these individuals had oral problems such as coating and food debris [30].

The theory of selective optimization with compensation (SOC) was developed to model adaptive development in a framework of the psychology of ageing, and describes how older people use different strategies to fulfil life goals and manage age-related declines [45]. SOC proposes that older people can accept age-related losses, and adapt and cope with changed function, by focusing on gains and strengths rather than losses and by finding ways to compensate for other limitations [46]. The three processes — selection, optimization, and compensation — have been investigated in a number of studies, mainly with a quantitative approach [47, 48]. Carpentieri and colleagues (2017) provided valuable knowledge about how older people themselves talked about their SOC strategies. Those who were engaged and talked about their SOC strategies had high level of well-being despite low physical function. Low well-being despite higher physical function were found among those who were little engaged in SOC strategies [49].

In Sweden, as well as in many other countries, the proportion of older people who have remaining teeth is growing. Thus, the demands for staffs’ knowledge and skills about oral care have increased. In order to understand how older people experience their oral health and their need for support with daily oral care while staying in a short-term care unit, qualitative studies are needed. Such knowledge might provide a deeper understanding that can improve nursing staff’s assistance with oral care. The aim of this study was therefore to describe how older people in short-term care experience their oral health and daily oral care.

## Method

### Design

A qualitative descriptive design was applied.

### Participants and setting

Three STC units in two Swedish regions were selected, representing both rural and urban areas, in different parts of Sweden. The units had 12–40 beds, and the staff consisted of 2–4 registered nurses, 12–40 licensed practical nurses, and one nurse aide. There were also occupational therapists and physiotherapists, either on site at the STC unit or visiting on certain days. The free-of-charge oral health assessment and staff education was not regularly performed at these STC units. Dental hygienist visits were performed only if the nursing staff detected oral health problems.

The inclusion criteria were that the participant should be 65 years or older, be able to communicate in the Swedish language, and have sufficient cognitive ability (based on patient records and judged by a registered nurse) to give informed consent to participate in an interview. The persons were old, and dependent on help with activities of daily living, and some older people where too frail to be able to participate. A purposeful sample of 15 persons (5 men and 10 women) were informed about the study and asked to participate and share their experiences of oral health and daily oral care. All 15 agreed to participate. One interview was excluded, due to inconsistent responses, thus the findings are based on 14 interviews. Some interviews were brief and did not contain rich narratives. However, the research team judged that no new information was obtained after 14 interviews.

### Data collection

The heads of social welfare services in the three municipalities were contacted and gave permission to collect data in the STC units. Following this, information was given to unit managers and nursing staff, and the nursing staff were asked to assist in selecting participants according to the inclusion criteria. Data were collected from November 2018 to December 2019 through individual semi-structured interviews conducted by the first author (S.K.). All participants received oral and written information about the study by the first author (S.K.) and gave their written informed consent.

An interview guide was developed (see supplementary file 1) for this study based on the researchers’ professional expertise in oral health, nursing, and older people care. The interview guide was first tested with an older person living in a nursing home and further pilot tested and revised. The opening questions concerned the participants’ general well-being and self-care ability, such as managing personal hygiene and getting dressed. These were followed by questions addressing their experiences of oral health and daily oral care, and if they received assistance with daily oral care. Participants who received assistance were asked questions about how they experienced the assistance, and participants who did not receive assistance were asked about reasons for not receiving assistance. All interviews were conducted in the participant’s room in the STC unit. The interviews were audio-recorded and lasted between 10 and 34 min. To gain richer descriptions, repeated interviews were conducted with two participants. Due to their frail state of health or their discharge from the STC unit, repeated interviews were not feasible for all participants.

### Analysis

Inductive qualitative content analysis was used as described by Elo & Kyngäs. This approach is suitable when the study is descriptive and there are few existing studies in the research field [50]. The analysis included several steps. In the **first step**, the interviews were transcribed verbatim by the first author. **Step two** was the preparation phase, when all authors read through the interviews individually to achieve a sense of the data as a whole. In **step three**, open coding was performed. Relevant text was highlighted and marked, thus creating meaning units that were then condensed and labelled with codes. In **step four**, three of the authors (S.K, E.C., and K.S.) discussed the codes and grouped similar codes into subcategories. Finally, in **step five**, all authors discussed and compared the subcategories, and abstracted similar statements into categories and a main category [50].

The analysis did not proceed linearly, but comprised a continuous process of moving between meaning units, original interview transcripts, codes, and categories. Before finalizing the categorization and the main category, all authors checked the elaborated findings for relevance regarding the meaning units, codes, and subcategories until consensus was reached. Finally, the presentations of the methods and findings were examined in a seminar with researchers and doctoral students.

### Ethical aspects

Older people in STC often have extensive health problems and may be vulnerable due to fatigue, impaired hearing, and impaired cognition. Many of those in STC may be experiencing a life crisis, not knowing whether they will be able to return home or whether they will have to move to special accommodation [12]. The study was planned according to the ethical principles of the Helsinki Declaration [51]. Asking questions about oral health and daily oral care can be ethically sensitive, and perceived as an intrusion to personal integrity. The participants were provided with oral and written information about the study, and gave their oral and written consent to participate. They were informed that their participation was voluntary, and that they could withdraw from the study at any time without an explanation and with no consequences for their care. Each transcript was given a code to ensure confidentiality and the data were stored so that no unauthorized person could gain access. The study was approved by the Regional Ethical Review Board in Uppsala, Sweden (ref: 2013/100/4).

## Results

The median age of the participants was 86 years (74–95 years). The participants were old, frail, and dependent on help with activities of daily living. Their characteristics are described in Table 1.

**Table 1:** Characteristics of the participating older people (*n* = 14) in short-term care facilities

Respondent	Sex	Age group	Reason for admission	Assisted daily oral care	Oral status
P1	Male	86–90	Respite care	No	Complete denture
P2	Female	81–85	Waiting for special accommodation placement	No	Remaining teeth without denture
P3	Female	76–80	Waiting for special accommodation placement	Yes	Remaining teeth without denture
P4	Female	76–80	Rehabilitation	No	Remaining teeth without denture
P5	Male	91–95	Waiting for home adaptation/modification	No	Remaining teeth, partial denture lower jaw
P6	Female	91–95	Waiting for special accommodation placement	No, but received assistance when admitted to hospital	Full denture upper jaw, partial denture lower jaw, remaining teeth
P7	Male	86–90	Waiting for special accommodation placement	Yes	Complete denture
P8	Female	91–95	Waiting for special accommodation placement	No	Remaining teeth without denture
P9	Female	71–75	Waiting for home adaptation/modification	No	Remaining teeth without denture
P10	Female	86–90	Rehabilitation	Yes	Remaining teeth without denture
P11	Female	71–75	Rehabilitation	No	Remaining teeth without denture
P12	Female	86–90	Respite care	No	Remaining teeth without denture
P13	Male	81–85	Waiting for special accommodation placement	No	Remaining teeth without denture
P14	Female	91–95	Waiting for special accommodation placement	Yes	Full denture upper jaw, remaining teeth

The participants’ experiences of oral health and daily oral care can be expressed as one main category: *Adapting to a changed oral condition while striving to retain independence*. This main category was built up from three categories and nine subcategories (Table 2).

**Table 2:** Overview of the main category, categories, and subcategories describing older people’s (*n* = 14) experiences of oral health and daily oral care in short-term care facilities

***Adapting to a changed oral condition while striving to retain independence***		
Wanting to manage daily oral care independently	Acceptance of changes in oral condition	Barriers to receiving assistance from staff
Having always brushed my teeth without help	Difficulty in chewing and swallowing	Staff lacking the time to help
Being satisfied with my mouth and teeth	Difficulty with tooth brushing	Not wanting to be a burden
Having to accept help if necessary	Not considering a dentist visit to be worth the cost	Lack of confidence in staff’s knowledge

### Wanting to manage daily oral care independently

This category was built up from three subcategories: *Having always brushed my teeth without help*, *Being satisfied with my mouth and teeth*, and *Having to accept help if necessary*. Overall, the participants described the importance to maintain control over tooth brushing for as long as possible, although they were well aware that their health was declining and their remaining lifetime was limited. They expressed satisfaction with their mouth and teeth, and gratitude for having teeth in old age. For many of them, the idea of receiving assistance with daily oral care was something they did not want to think about.

#### Having always brushed my teeth without help

Managing daily oral care was seen as important and well worth the effort. The participants expressed a strong desire to maintain their own management of daily oral care, as well as other daily routines. The participants considered brushing their teeth regularly once or twice a day to be a natural part of daily hygiene. Brushing their teeth was a lifelong and well-established habit that many wished to continue for as long as possible. It was difficult for them to imagine a situation where they no longer had the capability to maintain their oral hygiene, and difficult to accept the prospect of coming to the point when they would need to accept daily support with tooth brushing, as exemplified by one participant: *“I’d like to take care of it [tooth brushing] myself, and it’s worked out well for ninety years / … / so I must be happy”* (P8).

Another reason for wanting to perform tooth brushing without help was that the staff would come too early in the morning to assist, thus disturbing the participants’ sleep. The participants also noted that it would cause a sense of unease to have someone else’s fingers in their mouths. They said they were not sure if they could bear the thought of this, since they were used to performing oral care themselves. Strong feelings about the privacy of the mouth were expressed. Some also described a fear of provoking the gag reflex when reflecting on having to receive assistance with tooth brushing.

#### Being satisfied with my mouth and teeth

The participants were overall satisfied with their mouth and teeth, and did not experience pain in the mouth. They were grateful for still having their natural teeth in old age, even if the teeth had changed appearance and were not as white as before. Although they did not perceive having dentures as optimal, they felt that one learns to adapt with age. Being satisfied with their mouth and teeth was related to having functional teeth to be able to chew and eat: “*Yes, that’s the main thing, you can eat”* (P11).

#### Having to accept help if necessary

The participants commonly expressed that they “took one day at a time” and did not want to think about the days to come, when their ability to take care of themselves would decrease. Many stated that they could consider receiving assistance with daily oral care if they were no longer able to perform daily tooth brushing; as one participant stated: *“If you end up bedridden and unable to do anything by yourself, then it will be different”* (P7).

Some participants received regular assistance with tooth brushing, while some only received assistance occasionally. Such assistance could consist of help with brushing the teeth and dentures, or help with providing the utensils and applying toothpaste. Those receiving assistance had generally good experiences and were pleased with the oral care support they were given. They expressed that the staff were willing to assist them, and they felt that the staff considered this important. The participants described that accepting assistance with tooth brushing was beneficial for oral health in general, and that having clean teeth gave a nice feeling of freshness. This feeling also had a social aspect, since they saw it as unpleasant to expose themselves to others if they had unbrushed teeth.

### Acceptance of changes in oral condition

This category included three subcategories: *Difficulty in chewing and swallowing*, *Difficulty with tooth brushing*, and *Not considering a dentist visit to be worth the cost*. The participants described how their mouths and teeth had changed with older age, and one aspect of this change was the experience of chewing and swallowing problems. Tooth brushing was more difficult in old age and they perceived that it would not be worth the cost to repair broken teeth or old dentures.

#### Difficulty in chewing and swallowing

Dry mouth was particularly experienced during the night, and so the participants kept a cup of water beside the bed. Participants with dentures described difficulties with chewing certain foods, and that they could not chew the bolus as fast as they had been able to with their natural teeth. Bread was easier to chew than meat. In addition, the meal components had to be cut into smaller pieces, especially meat. Some of the participants who wore dentures expressed that their dentures were poorly fitting, which resulted in a feeling of the denture “falling down” from the upper jaw when eating or speaking. They also found that the teeth of the dentures were not as sharp as their natural teeth, which affected their ability to chew. Those with broken teeth were not able to chew correctly because it felt as if their teeth were going to fracture. One participant had to be particularly careful to chew food carefully before swallowing, to avoid the bolus getting stuck in her throat: *“You have to chew properly and not swallow too fast, it feels like it gets stuck in your throat, but it is manageable*” (P6).

#### Difficulty with tooth brushing

Participants with natural teeth explained that they had some broken teeth that made it difficult for them to clean their teeth. Food debris between the teeth was hard to remove, and they described a feeling of having a coating on their teeth. Tooth brushing could also be difficult due to reduced vision or impaired balance. Having remaining teeth and not being able to take care of them as before gave them a feeling of fear of losing their teeth. Other reasons for not having the power to take care of oral hygiene included lack of motivation caused by remaining fatigue after acute illness and hospital care, and long periods of depression. Tooth brushing had been easier to perform when they were younger, but became more difficult with increasing age, as expressed by one participant: *“It’s not like when you were young … they [the teeth] were easier to take care of, or you took care of them better then”* (P2).

#### Not considering a dentist visit to be worth the cost

In the participants’ younger years, regular dental visits had been more or less part of daily life. Some still saw regular dental visits as important, while others had stopped visiting dental care regularly. Long hospitalization, declining overall health, and lack of energy made it difficult to visit dental care. In general, dental visits had decreased since retirement, and most participants saw toothache as the only reason for an urgent visit to the dentist. Dental care remained a low priority for participants who described their dentures as poorly fitting and insufficiently sharp for proper chewing.

The participants considered repairing broken teeth or old dentures to not be worth the cost, as they felt that given their old age and limited remaining lifespan they could manage with their current oral state: *“But life is so short... that it can’t be worth doing anything to fix it... I’ll do fine with what I have”* (P1).

Many participants had not visited the dentist for many years because they did not think it was important, as long as they did not have toothache. They also described that they had not suffered from aching teeth for many years, although this was something they had experienced when they were young. Some participants did not think that their teeth were important anymore and stated, like this participant, that it was more important to avoid toothache than to retain many teeth: *“Now I don’t think it’s so important to have lots of teeth left. The main thing is that they don’t ache or anything”* (P8).

### Barriers to receiving assistance from staff

This category contained three subcategories: *Staff lacking the time to help*, *Not wanting to be a burden*, and *Lack of confidence in staff’s knowledge.* Barriers to receiving assistance with daily oral care were described as a feeling of staff lacking time to help and a fear of becoming a burden if they asked for help with daily oral care. Moreover, they expressed a lack of confidence in the staff’s knowledge and skills in assisting with tooth brushing.

#### Staff lacking the time to help

Participants described a feeling of stress when the staff member was standing by the door, observing the participant as they brushed their teeth and waiting for them to finish. This stressed situation gave a feeling of not having enough time to clean their teeth as thoroughly as desired. Overall, the participants perceived the staff as being too busy to assist with daily oral care, though some participants felt they could consider asking for assistance if the staff had the time. They thought that the staff had a hard workload: *“The staff are expected to perform as much as possible in the shortest possible time”* (P4).

#### Not wanting to be a burden

One reason for not asking for assistance with tooth brushing and other oral care was that the participants were afraid the staff would feel they were nagging them. The participants did not want to be a burden and interfere with the work of staff who were busy with other tasks. Some expressed empathy with the staff, believing that they had other work tasks to do that were more important than assisting with oral care: “*… they certainly have more important things to do than to brush my teeth … I’d probably see it as inconvenient for them to do it”* (P13).

#### Lack of confidence in staff’s knowledge

Participants expressed a lack of confidence in the staff’s knowledge and skills in assisting them with tooth brushing. They described how they wanted to take care of their teeth in a proper manner according to their own habits, because they were uncertain if the staff would know how to brush their teeth in the way they preferred. One participant would ask her family members for help with tooth brushing rather than asking the staff, if she lost the ability to brush her own teeth. The participants had different levels of confidence in different staff, as expressed by one participant: *“Some you have more confidence in than others, that’s how it is”* (P4).

## Discussion

To our knowledge, this is the first study describing older people’s experiences of oral health and assisted daily oral care in a STC context. Older people admitted to STC are in transition, and may experience this as an uncertain period, not knowing if they will be able to return home or will have to move to special accommodation. This period of their lives could have a negative impact on their oral health, and so exploring their experiences of oral health and daily oral care is important.

We will discuss these older people’s experiences of oral health and daily oral care using the theory of SOC [45], and will also examine barriers to receiving assisted daily oral care.

The main category in our findings was *Adapting to a changed oral condition while striving to retain independence*. The participants’ strong desire to continue performing daily oral care by themselves could be the result of selecting an activity they can still perform and that gives them satisfaction. Moreover, continuing to work at maintaining a certain activity, such as daily tooth brushing, is an example of optimization as described in SOC [45]. Not wanting assistance with daily oral care can be interpreted as the participants’ choice to maintain some degree of independence despite needing compensation via assistance from staff to manage personal hygiene, dressing, and mobility. The participants were able to imagine having assistance with oral care if they were no longer capable of performing oral care themselves, which is another example of compensation.

Other studies conducted among older people found that selection of activities was focused on maintaining activities of daily living as independently as possible [52, 53]. Participants in the present study described how they had adapted to changes in oral health. Such descriptions may be related to their having experienced dental visits as not being of particular importance, or being considered as too expensive. Another obstacle may be lack of energy, forcing the participants to select between a visit to dental care and other, more valued, activities. Participants with dentures described having adapted to their dentures; they prioritized proper chewing, and compensated for their chewing difficulties by cutting their food into small pieces. Similar strategies were described in a study by Khabra and colleagues (2017) conducted among community dwelling older people who received rehabilitation and nursing assistance. The participants in that study described how they used SOC strategies, optimization, and compensation to adapt and manage their changing dental status. They had adapted to their dentures over time, and had modified their food choices and chewing accordingly [54].

One reason for the perceived satisfaction with oral condition despite deteriorated function might have been that the respondents were comparing their oral conditions with those of previous generations who often had dentures or were edentulous. Earlier research has found that older people may adapt to oral disorders, and that adaptation usually involves comparison with other disabilities such as chronic disorders and functional limitations [38, 55]. The participants in this study were old and frail, and may have had other medical, personal, and social challenges which could have made them experience their oral problems as less important than other health problems. This would be in line with the findings of Donnelly and colleagues, who studied the relationship between perceived oral health, body image, and social interactions among older people living in long-term care facilities [38]. Another study exploring frail older people’s experiences of health found that those who perceived their symptoms and disorders as manageable were more likely to experience safety and control, and hence to also experience good health [56].

Older people’s desire to stay independent for as long as possible and to take care of themselves has been described in previous studies [39, 57, 58]. Being independent and having the ability to perform activities of daily living contributes to a sense of control [58] and influences older people’s perceived health and well-being [57, 59]. Similarly to our findings, Niesten and colleagues (2012) found that older people wanted to be responsible for taking care of their own teeth. The researchers suggested that the participants’ sense of control could be related to experiences of autonomy and independence, which were important factors for their quality of life [59]. Being able to perform daily activities contributes to a sense of control and autonomy; the ability to manage on one’s own means being able to maintain dignity and not feel like a burden to others [58]. For many people, being in STC is a new situation, and a place where they expect to be for only a limited period. They might arrive from their own home, where they were used to performing daily tasks like tooth brushing themselves. Daily oral care may be one of the activities they feel that they can continue doing independently even if they are dependent on assistance with other daily activities. This is an example of how individuals select those activities that are most realistic in relation to their resources [46].

Barriers to accept necessary assistance could lie both with the older person and the nursing staff [6]. The participants in this study mainly expressed these barriers as wanting to manage daily oral care independently. This striving to maintain some degree of independence for as long as possible has also been found in previous studies [37, 39]. However, such striving may also be a barrier for the older person to accept assistance with daily oral care when needed. Another barrier to getting assistance was that the participants felt that the staff had many duties, and they did not want to nag or be a burden to the staff. Similar findings have also been described in recent studies [39, 60, 61]. One explanation for not wanting to burden the staff could be that when older people are used to managing their bodily care by themselves, without having to take another person’s availability into account, it can be difficult to acknowledge and accept the need for help [58].

Some participants were uncertain if the staff would know how to brush their teeth in the same way as they preferred and expressed a lack of confidence in the staff’s knowledge and skills to assist them with oral care. This lack of confidence could also be a barrier to accepting assistance from staff. Low prioritization of oral care due to staff’s lack of knowledge and time has previously been described from the perspective of the staff [26, 43]. Nursing staff’s lack of knowledge about providing oral care caused feelings of inadequacy and insecurity, and the staff expressed a need for continuous education in the field. They also considered it difficult to ask for permission to assist with oral care because the mouth was seen as private and intimate [43]. Although bodily care is the heart of nursing, nursing staff may not be comfortable in providing such care, including brushing older people’s teeth’s [40] which may reflect a lack of oral health education [41].

Participants in the present study described a feeling of stress and not being given enough time to complete their oral care when the staff were standing by the door, waiting while the participants performed oral care. Older peoples’ wishes to maintain independence in managing oral care need to be acknowledged but should be balanced by a judicious provision of oral care support. It is therefore important to identify older people’s individual oral health needs and wishes, and to ask about assistance with daily oral care in a person-centred way. For this reason, it is important to have a close collaboration between dental and nursing staff [27], and to provide education in oral health, including clinical assessment of oral care, for health care staff.

The participants in this study lacked regular dental care, and did not think it was worth the cost or effort to visit the dental clinic. This may be due to their declining overall health, and the presence of other major changes in their lives; for example, some of them were waiting for transfer to special accommodation. Thus, it is especially important to ensure that people in STC entitled to a free-of-charge oral health assessment and “reduced-cost dental care” [16] actually receive those services.

### Limitations and final considerations

Data were collected during more than 1 year, from November 2018 to December 2019. This was due to problems with recruiting participants according to the research plan; that is, people who received assistance with their oral care. The problems could be explained by two circumstances. Firstly, STC is a context where people are in transition; on their way home or to special accommodation, or receiving end-of-life care. Secondly, those potential participants who received assistance with oral care were frail and often had cognitive difficulties or other impairments. Thus, the voices of those who received assistance with oral care were not as prominent as the voices of those who managed independently.

People aged 80 years and older who are in need of daily care often have some cognitive or communicative impairment, and may experience memory lapses or difficulty with word recall, but they are often able to participate in interviews [62]. Interviewing can be tiring for frail older people; some of our participants had to lie down and rest after less than 30 min of interviewing. The participants’ situation might also have had an effect on the interviews, as it might have been difficult to engage in a research interview while accepting one’s deteriorating health and worrying about having to leave one’s own home.

The interviewer has a background as a registered dental hygienist and is familiar with working with older people’s oral health. Most interviews were short and not rich in information, most likely due to the situation described above. The skills of the interviewer are important for the quality of interviews, and these skills developed during the data collection. Such experience has also been described by other researchers performing interviews with older people [39]. Oral health and daily oral care could be perceived as an intimate issue, and something that many people did not feel comfortable talking about. Data that are not rich in descriptions of the participants’ experiences can threaten the trustworthiness of a qualitative study. One strategy that can be used is to perform repeated interviews with the participants. It was difficult to do this in the STC context, as participants moved out from the STC, were admitted to hospital care, or even died.

Since we are just starting to explore an important phenomenon for a growing population, we believe that our findings can provide valuable knowledge for the care of frail older people. However, there is a need for more studies, and interviews combined by observational studies [63] as well as an ethnographic design might provide deeper knowledge.

Other aspects of trustworthiness should also be considered. Dependability and confirmability were achieved by ensuring that all researchers were involved in all steps of the analysis process [50]. Credibility was addressed by scrutinizing the study in a multi-disciplinary research seminar [64]. Moreover, the researchers who conducted the study had different professional perspectives on oral care (registered nurses and registered dental hygienists). A detailed description of the research process, settings, and participants is provided to address transferability.

## Conclusion

The participants in this study were generally satisfied with their oral health and wanted to manage their daily oral care independently for as long as possible. However, they were willing to consider assistance with daily oral care when they got to the point of not being able to perform this themselves. They described both a need for assistance and barriers to such support. Based on our findings, there is a need to increase nursing staff’s attention towards older peoples’ oral health and their ability to assist older people with daily oral care.

Clinical practice guidelines for oral health care of older people and their specific need should be present. To allow follow-up and feedback to health care staff, data on oral status, needs for assisted oral care and outcomes should be systematically collected and used to support development of care.

Health care staff in short term care settings should be provided with education and training in assessing and performing oral care. Person-centered care should be an intrinsic part of such education. In addition, continuous follow-up and feedback on oral care practice needs to be implemented, including the perspectives of older people.

## Supplementary Information

**Additional file 1.** Interview guide.

## Data Availability

The interview dataset generated and analysed during the current study are not publicly available due to an agreement with the participants on the confidentiality of the data. However, the data could be available from the corresponding author on reasonable request.

## Abbreviations

SOC: The theory of selective optimization with compensation
STC: Short-term care
