# Supplementary material for: Older people’s experiences of oral health and assisted daily oral care in short-term facilities
Source: BMC Geriatr. 2021 Jun 27;21:388. doi: 10.1186/s12877-021-02281-z (PMC8237451; doi:10.1186/s12877-021-02281-z)
Supplement: Supplementary file 1 — Additional file 1. Interview guide. [file 12877_2021_2281_MOESM1_ESM.docx]

**Interview guide**

**Initial questions**

How are you feeling right now, in general?

Can you manage your personal hygiene, for example washing and getting dressed?

**Experiences of oral health**

**Probes**

Could you describe more….

How did you experience…..

In what way….

What did you feel……

Describe how you experience your mouth and teeth?

Has anything changed with your mouth and teeth during the last year?

Are your teeth and your mouth important to you?

**Daily Oral care**

How do you currently take daily care of your teeth and mouth?

Do you need help with taking daily care of your teeth and mouth?

Do you receive assistance with daily oral care?

**Those receiving assistance with daily oral care**

How do you perceive it to get help with your daily oral care?

**Those not receiving assistance with daily oral care**

What are the reasons for not receiving assistance (personally, staff)?

If you imagine a situation when you really need assistance with daily oral care, how do you think you would experience that?

Is there anything else that you would like to share about your oral health that we haven’t discussed?
